# Dysregulated Phosphorylation of p53, Autophagy and Stemness Attributes the Mutant p53 Harboring Colon Cancer Cells Impaired Sensitivity to Oxaliplatin

**DOI:** 10.3389/fonc.2020.01744

**Published:** 2020-08-28

**Authors:** Lubna Therachiyil, Javeria Haroon, Fairooz Sahir, Kodappully S. Siveen, Shahab Uddin, Michal Kulinski, Joerg Buddenkotte, Martin Steinhoff, Roopesh Krishnankutty

**Affiliations:** ^1^Translational Research Institute, Academic Health System, Hamad Medical Corporation, Doha, Qatar; ^2^Department of Pharmaceutical Sciences, College of Pharmacy, Qatar University, Doha, Qatar; ^3^Department of Dermatology and Venereology, Hamad Medical Corporation, Doha, Qatar; ^4^Department of Medicine, Weill Cornell Medicine-Qatar, Qatar Foundation-Education City, Doha, Qatar; ^5^Department of Medicine, Weill Cornell Medicine, New York, NY, United States; ^6^College of Medicine, Qatar University, Doha, Qatar

**Keywords:** colon cancer, HCT 116, HT 29, p53, oxaliplatin, autophagy, therapy, drug resistance

## Abstract

Colorectal cancer (CRC) forms one of the highest ranked cancer types in the world with its increasing incidence and mortality rates despite the advancement in cancer therapeutics. About 50% of human CRCs are reported to have defective p53 expression resultant of *TP53* gene mutation often contributing to drug resistance. The current study was aimed to investigate the response of wild-type *TP53* harboring HCT 116 and mutant *TP53* harboring HT 29 colon cancer cells to chemotherapeutic drug oxaliplatin (OX) and to elucidate the underlying molecular mechanisms of sensitivity/resistance in correlation to their p53 status. OX inhibited growth of wild-type p53-harboring colon cancer cells via p53/p21-Bax mediated apoptosis. Our study revealed that dysregulated phosphorylation of p53, autophagy as well as cancer stemness attributes the mutant p53-harboring colon cancer cells impaired sensitivity to OX.

## Introduction

Colorectal cancer (CRC) is ranked as the third most commonly diagnosed cancer type after the lung and breast cancers worldwide beyond its geographical variations and localized temporal trends (1). The global burden of CRC is expected to increase due to the huge rise in its yearly incidences around the world making it the fourth cause of cancer deaths worldwide (2). Despite the advancements in pharmacokinetics with introduction of novel therapeutics, nearly half of the CRC patient population is reported to have the disease relapse (3, 4). Chemoresistance, a feature by which the cancer cells become less sensitive to chemotherapeutic drugs forms one of the major reason for this disease relapse and pose a major challenge in cancer treatment regime (5). The factors contributing to chemoresistance are many, with cancer stemness, impaired drug transport as well as targeted gene mutations being the most highlighted ones (6). Though mutations in several genes has been extensively correlated to CRC development, *TP53* (p53) forms one of the most commonly mutated genes in cancer (7). About 50% of CRC cases are known to pose this mutation (8). An impaired cross talk between mutant p53 and its apoptotic target genes with no transactivation and enhanced tumorigenic properties such as survival, proliferation, angiogenesis, and chemoresistance has been reported to contribute to CRC disease progression (9, 10).

p53 is regarded to be a tumor suppressor transcription factor, which upon stress particularly DNA damage inducing stimuli gets stabilized by posttranslational modifications primarily *via* phosphorylation of serine and threonine residues (11). This stabilization transactivates several target genes that regulates various cellular functions including cell cycle, apoptosis, senescence, DNA repair, and cellular metabolism which functionally leads to tumor suppression (12). Cancer related p53 mutations can lead to loss of its function as tumor suppressor while is reported to acquire a function termed as gain-of-function (GOF) that enriches the mutant p53 with oncogenic properties which promotes tumorigenesis (13, 14). With GOF, mutant p53 not only gets dominated with its pro-oncogenic properties but also, facilitates drug resistance (15, 16).

Oxaliplatin (OX), also known as Eloxatin is a third generation platinum based chemotherapeutic agent widely used in clinics for the treatment of CRC (17). Structurally, OX has a core platinum atom bound to an oxalate group-also called as the displacement group and diaminocyclohexane (DACH) group-which is a carrier ligand (18). OX is classified as an alkylating agent that exerts its cytotoxicity effects *via* introducing DNA damage in target cells (19). OX is known to produce DNA mono/di-adducts *via* forming DNA intra-/inter-strand crosslinks as well as DNA–protein crosslinks (20). DNA di-adducts formed by binding to N (7) site of the guanine are fatal, can lead to DNA lesion further inducing G2/M phase arrest or apoptosis (21). Despite its efficient mode of action, most of the cancer cells apart from its primary sensitivity to OX is found to ultimately acquire resistance (17). Most of the *in vitro* studies on the OX resistance in colon cancer cells are carried out in stable resistantly raised cell lines (17, 22).

The current study aimed to identify any existing link between p53 status of colon cancer cells and their sensitivity towards the chemotherapeutic drug OX. The response to genotoxic stress induced by OX in human derived HCT 116 (wild-type p53) and HT 29 (mutant p53) colon cancer cell lines were evaluated using various biochemical assays and the underlying molecular mechanisms were elucidated.

## Materials and Methods

### Chemicals

The drug OX used in this study was purchased from Sigma Aldrich (St. louis, MO, United States). The solid drug was dissolved in dimethyl sulfoxide (DMSO) to a stock concentration of 25 mM. The stock was aliquoted and stored in −20°C and diluted in cell culture media to desired concentration(s) for drug treatment experiments. Chloroquine Diphosphate salt was purchased from Sigma Aldrich (St. louis, MO, United States) and dissolved in sterile water to a stock concentration of 19 mM, further diluted in cell culture media to prepare working stock of 10 μM.

### Cell Lines and Culture Methods

The two human derived CRC cell lines used in the study were acquired from the American Type Culture Center, ATCC (Manassas, VA, United States). Cell lines: HCT 116 and HT 29 were cultured in Dulbecco’s modified Eagle’s medium (DMEM). The culture media was supplemented with 10% (v/v) Fetal Bovine Serum (FBS) and 1% antibiotics cocktail (penicillin 100 μg/ml, and streptomycin 10 μg/mL), grown at 37°C in a humidified atmosphere of 5% CO_2_ and 95% air.

### Cell Cytotoxicity Analysis

Cytotoxicity assay was performed using Cell Counting Kit – 8 (CCK-8; Sigma-Aldrich, St. louis, MO, United States) as per the manufacturer’s instructions. Briefly, cells (5000 cells/well) were seeded into 96-well plates and allowed to adhere for 24 h. The cells were treated with either OX (20 μM) or vehicle (DMSO) alone for the indicated time periods. For inhibitor studies, cells were pre-treated with autophagy inhibitor chloroquine (CQ; 10 μM) for 1 h followed by treatment with OX (20 μM) for the indicated time periods. After the desired periods of drug treatment, 10 μL of CCK-8 reagent was added to the culture media and incubated at 37°C for 2 h. The absorbance was read at 450 nm in a plate reader. The growth inhibition was calculated and expressed as percent of the vehicle control.

### Morphological Changes

To investigate the morphological changes with OX treatment, the cells (5 × 10^5^ cells/well) were seeded into 6-well plates and incubated overnight at 37°C (with 5% CO_2_) to allow the cells to adhere. The cells were then treated with 20 μM OX and imaged at 0, 24, and 48 h posttreatment under the bright field microscope (Nikon ECLIPSE, Tokyo, Japan) at 20 × magnification.

### Apoptosis Assay

Apoptosis assay kit Annexin-V-FITC/PI (BD Biosciences, San Jose, CA, United States) was used to measure the apoptotic activity within the cells. The kit was used according to the manufacturer’s instructions. In brief, the cells after overnight adherence were treated with 20 μM OX. At 0, 24 and 48 h posttreatment the cells were collected by trypsinization, washed and suspended in binding buffer. The cells were then stained and analyzed by flow cytometry using a BD LSRFortessa analyzer (BD Biosciences) as reported earlier (23). The data was quantified and expressed as percent of the cell counts.

### Bax/Bcl-2 Quantification

The western blot analysis were standardized for repeatability and reliability by performing the protein quantification using bicinchoninic acid assay (BCA) gold kit as well as loading equi-amount of protein (40 μg per lane) into the gel. The transfer efficiency was routinely checked by Ponceau Red staining of the membranes as well as setting the same exposure time for chemiluminescent detection. The linearity of protein expression of Bax and Bcl-2 were initially checked using standard in-house cell extracts and optimized for the dilution of antibodies. The Bax/Bcl-2 protein expression was quantified by densitometry analysis using Image Lab Software V6.1 (Bio-Rad, Munich, Germany). Values of protein expression in arbitrary units were normalized to the loading control (GAPDH) and represented as relative ratio.

### Phosphoprotein Array Analysis

Cell lysates prepared from OX treated or untreated cells at 48 h post treatment were analyzed for phosphorylation of proteins using Proteome Profiler Human Phospho-Kinase Array (R&D systems, Minneapolis, MN, United States) according to the manufacturer’s instructions. In brief, cell lysates from untreated and treated cells were prepared using lysis buffer (containing protease and phosphatase inhibitor cocktails) supplied with the kit and protein concentration estimated by Rapid Gold BCA Protein assay kit (Pierce^TM^, Thermo Scientific, Waltham, MA, United States). Cell lysates diluted in array buffer were incubated with the ready-to-use pre-coated array membranes (blocked in blocking buffer provided with the kit) overnight at 4°C on a rocking platform shaker. The array membranes were further processed as described in Leo et al. (23) and visualized using a ChemiDoc^TM^ MP imaging system (BioRad, Hercules, CA, United States). The pixel density of each phosphorylated protein spots (in duplicates) in array were averaged and normalized against the reference spots and the relative levels were expressed as mean pixel intensity. Array coordinates and identities of all antibodies are detailed in the Supplementary Material.

### Western Blot Analysis

The cells were treated with 20 μM OX for indicated time periods and lysed in cold radioimmunoprecipitation assay (RIPA) buffer supplemented with 1x protease-phosphatase cocktail inhibitors (Roche). The protein lysate was clarified by centrifugation at 14,000 × *g* for 15 min at 4°C. Western blotting was performed as described previously (23). The immunoblotting was performed in whole blot membrane independently against each antibodies: Bax, Bcl-2, p53, p21, LC3-I/LC3-II, CD133, CD44, β-catenin, and GAPDH. All the antibodies were purchased from Cell Signaling Technologies (Beverly, MA, United States).

### Statistical Analysis

Statistical analysis was carried out using the software GraphPad Prism 8.0 and significance calculated using non-parametric Welch’s *t*-test. A *p*-value < 0.05 was considered statistically significant. Data are represented as mean ± SD from three independent experiments unless otherwise stated.

## Results

### Oxaliplatin Significantly Inhibited Growth of Colon Cancer Cells With Wild-Type p53 Than With Mutant p53

**FIGURE 1:**
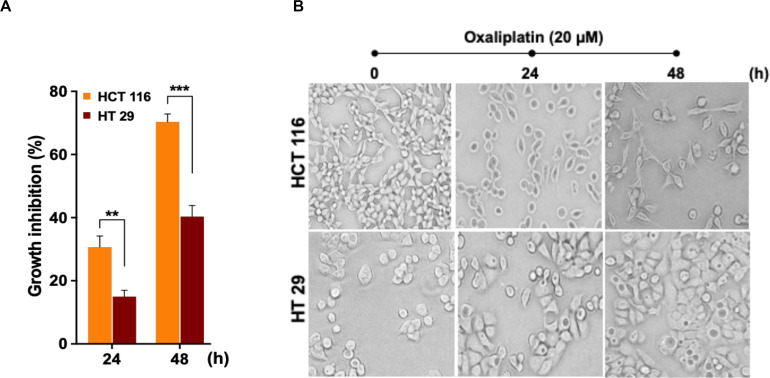
Oxaliplatin (OX) significantly inhibited the growth of HCT 116 cells. **(A)** Percentage growth inhibition of time-course treatment of HCT 116 and HT 29 cells with OX (20 μM) as estimated using CCK8 kit. Data represented as mean ± SD (*n* = 6). Significance was calculated using Welch’s *t*-test; **p = 0.007, ***p = 0.0005. **(B)** Effect of OX treatment on morphology of colon cancer cell lines. OX treatment resulted in significant growth inhibition of HCT 116 cells with much reduced cell confluency compared to HT 29 cells. Images were captured using bright field microscope (Olympus IX51, objective 20x).

In order to assess cytotoxic effects of the drug OX on colon cancer cells in correlation to their p53 status, the wild-type p53 bearing HCT 116 cells and the mutant p53 bearing HT 29 cells were treated with OX (20 μM) and their cytotoxicity were measured by CCK8 assay after 24 and 48 h. A time-dependent growth inhibition was observed in both the cell lines with the OX treatment. Comparatively, the growth inhibition was significantly higher in wild-type p53-HCT 116 cells accounting to 70% after 48 h of OX treatment, while in mutant p53-HT 29 cells the growth inhibition accounted to only 40% (Figure 1A). This differential growth inhibition exhibited by the colon cancer cells to OX in the context of their p53 status implied that, HCT 116 cells bearing wild-type p53 was sensitive to OX treatment while HT 29 cells with mutant p53 were resistant or less sensitive to OX treatment.

The differential growth inhibition in both the colon cancer cells treated with OX were further evidenced from their morphological changes. The microscopy images of the cells before and after treatment with OX showed a significant growth inhibition in HCT 116 cells with much reduced confluency while, in the case of HT 29 cells no such morphological changes were observed with cells found to be confluent and viable even in the presence of OX (Figure 1B).

### Oxaliplatin Induced Apoptosis via Bax Expression and Bcl-2 Repression in Wild-Type p53 Colon Cancer Cells

**FIGURE 2:**
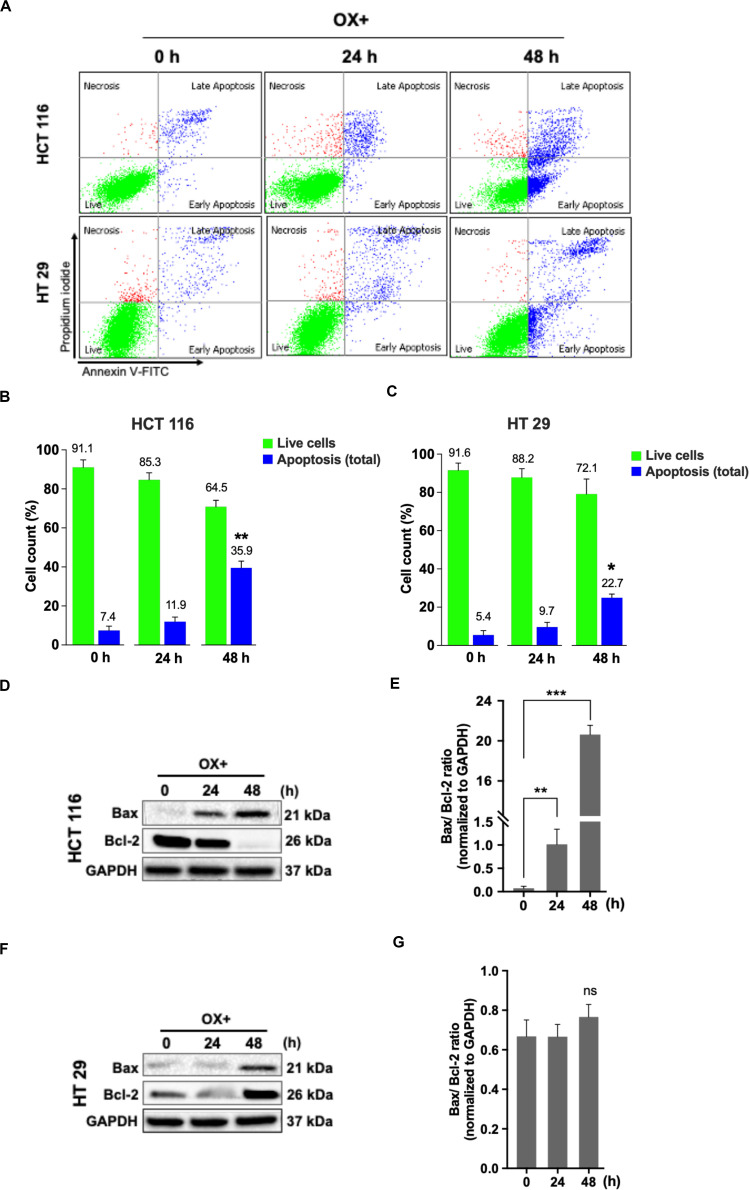
OX induced apoptosis *via* Bax expression and Bcl-2 repression in HCT 116 cells. **(A)** Dot plots showing the distribution of cell population in quadrants as indicated and are representative of three replicate experiments. **(B,C)** Percent distribution of live cells and apoptosis at 0, 24 and 48 h of HCT 116 **(B)** and HT 29 cells **(C)**. Significance was calculated using Welch’s *t*-test; *p < 0.05, **p < 0.001. **(D,F)** Western blot images showing the Bax and Bcl-2 protein expression. GAPDH served as the loading control. **(E,G)** Ratio of the protein levels quantified by densitometry analysis. **p = 0.007, ***p < 0.0001. The images are representative of three independent experiments and data represented as mean ± SD (*n* = 3).

Apoptosis being a prominent cell death mechanism of growth inhibition in cancer cells under genotoxic stress, we tried to measure the apoptosis events in the colon cancer cells treated with OX. The cells were stained using Annexin-V-FITC/PI kit and analyzed by flow cytometry. OX induced apoptosis in both the colon cancer cells (Figure 2A) while, the percent apoptosis was found to be significantly higher in HCT 116 cells after 48 h of treatment compared to HT 29 cells (Figures 2B,C). The Bax/Bcl-2 ratio functions as a rheostat that ascertains the cell defenselessness toward apoptosis (24). Elevated Bax/Bcl-2 ratio levels has been proven to drive human cancer cells to apoptosis as well as reduce tumor aggressiveness (25). A time-dependent increase in Bax/Bcl-2 ratio was observed in HCT 116 cells with OX treatment (Figures 2D,E) while in HT 29 cells, with Bax repression and BCL-2 expression, no significant difference in this ratio was observed with OX treatment (Figures 2F,G). The non-modulated Bax/Bcl-2 ratio in mutant p53 HT 29 cells in response to OX treatment could be attributed to their impaired sensitivity to the drug.

### Wild-Type p53 Bearing Colon Cancer Cells Sensitivity to Oxaliplatin Was p53/p21 Mediated

**FIGURE 3:**
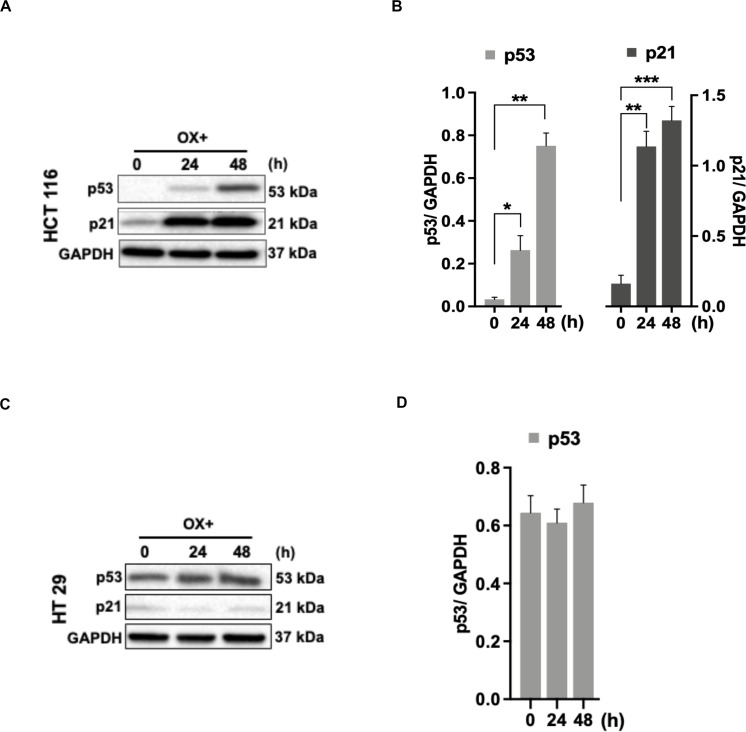
p53/p21 complex mediated sensitization of HCT 116 cells by OX. **(A,C)** Western blot images showing the expression of p53 and p21. GAPDH served as the loading control. **(B,D)** Relative protein levels quantified by densitometry analysis. Significance was calculated using Welch’s *t*-test; *p = 0.02, **p < 0.001, ***p < 0.0002. The images are representative of three independent experiments and data represented as mean ± SD (*n* = 3).

As the functional mechanism of wild-type p53 expression is known to be mediated by the activation of its primary effector target p21 – a cyclin-dependent kinase inhibitor (CDKi) (26), we sought to determine the p21 expression in colon cancer cells in response to OX treatment in the context of p53 status. A concomitant increase in p21 protein expression along with p53 expression in response to OX treatment was observed in HCT 116 cells (Figures 3A,B), while OX treated HT 29 cells did not induce any p21 protein expression wherein, the p53 protein expression was found to be endogenous (Figures 3C,D). This data indicates the positive role of p53/p21 complex in sensitizing wild-type p53 HCT 116 cells to OX and the lack of functionality of this complex formation in mutant p53 HT 29 colon cancer cells makes them less sensitivity to OX treatment.

### Oxaliplatin Induced Phosphorylation of N- and C-Terminal Serine Residues of Wild-Type p53, Not Mutant p53

Phosphorylation, a key posttranslational modification is known for its potent role in rendering wild-type p53 protein’s functionality as a tumor suppressor (27). In particular, the stabilization and activation of p53 in response to genotoxic stress induced by platinum-based therapeutics are reported to be facilitated *via* phosphorylation of the serine residues (28, 29). Based on these reports, we intended to determine the phosphorylation of N-terminal and C-terminal serine residues of p53 protein in wild-type p53 bearing HCT 116 and mutant p53 bearing HT 29 colon cancer cells in response to OX treatment.

**FIGURE 4:**
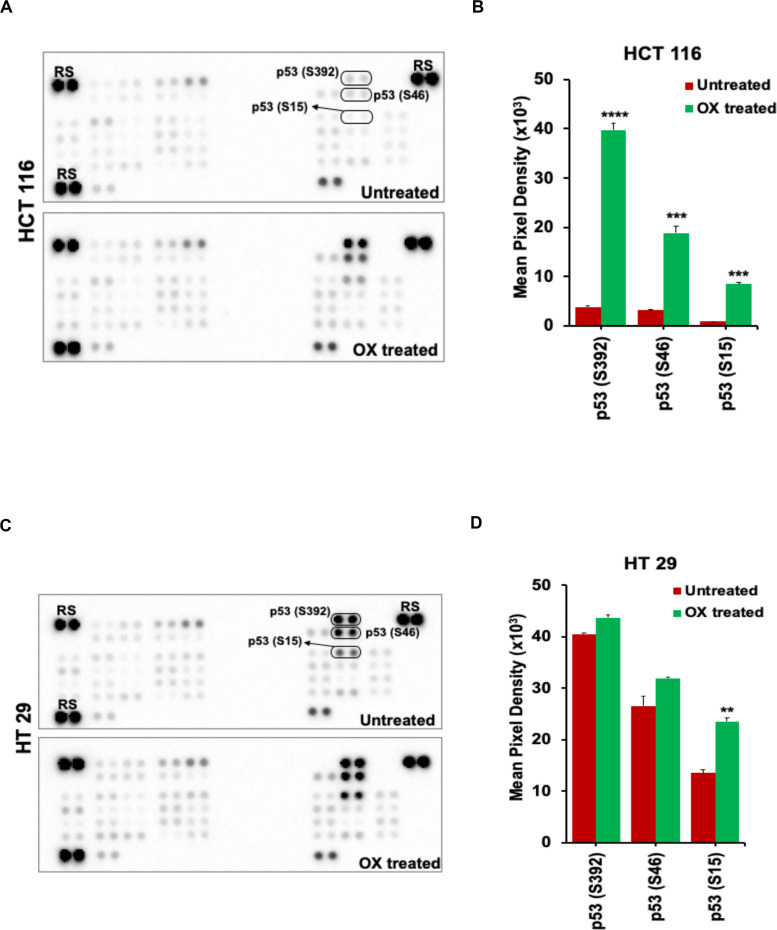
OX induced phosphorylation of N-terminus and C-terminus serine residues of wild-type p53. **(A,C)** Representative images of the antibody arrays showing phosphorylation levels of serine motifs (as indicated) of p53 protein analyzed using Proteome profiler Human Phospho-Kinase Array (R&D Systems). **(B,D)** Quantitative profiles of phosphorylation levels of serine motifs on antibody arrays by densitometry analysis. Significance was calculated using Welch’s *t*-test; **p = 0.002, ***p < 0.0001, and ****p < 0.0003 Values are represented as mean ± SD (*n* = 3). Images are representative of three independent experiments. RS denotes reference spots. The arrays are spotted with the antibodies in duplicates. The array coordinate map with the corresponding antibody/protein names are as illustrated in Supplementary Figure S1.

The OX treatment was found to induce phosphorylation of N-terminal serine residues: S15, S46, and the C-terminal serine residue: S392 of the wild-type p53 in HCT 116 cells. This was evidenced from the significant increase in spot intensities within the antibody array (Proteome Profiler^TM^ Human Phospho-Kinase antibody Array) incubated with the lysate of OX treated HCT 116 cells compared to the untreated (Figures 4A,B). In the case of HT 29 cells, phosphorylation of S15, S46, and S392 of the mutant p53 was observed to endogenous (Figures 4C,D) indicating that the phosphorylation of mutant p53 was non-inducive and independent of OX treatment. This data, an indicative of dysregulated phosphorylation event in mutant p53 could be correlated to the prominence of p53 status in attributing sensitivity to OX. The wild-type p53 got stabilized and became functionally active by phosphorylation of serine residues in response to the genotoxic stress thereby sensitizing the wild-type p53 bearing colon cancer cells to OX. The endogenous phosphorylation of serine residues of mutant p53 protein, an unfavorable feature for stabilization/activation pursued toward a functional p53 could be attributed to the impaired sensitivity to OX displayed by the mutant p53 bearing colon cancer cells.

### Autophagy Attributes the Mutant p53 Harboring Colon Cancer Cells Impaired Sensitivity to Oxaliplatin

The cancer cells are known for their self-eating capability – a cell death process known as autophagy to overcome metabolic stress which forms a pro-survival mechanism (30). Studies has proven autophagy as one of the cytoprotective mechanisms adapted by cancer cells to overcome genotoxic stress thereby making the cells resistant to chemotherapeutic drugs (31). In our study, we looked for any induction of autophagy in colon cancer cells in response to OX treatment. The microtubule-associated protein 1A/1B-light chain 3 protein (LC3) and its conversion from LC3-I form to LC3-II is recognized as a hallmark of autophagy induction, as LC3-II forms the surface protein marker of autophagosomes: the lysosomal vesicles formed during autophagy (32).

**FIGURE 5:**
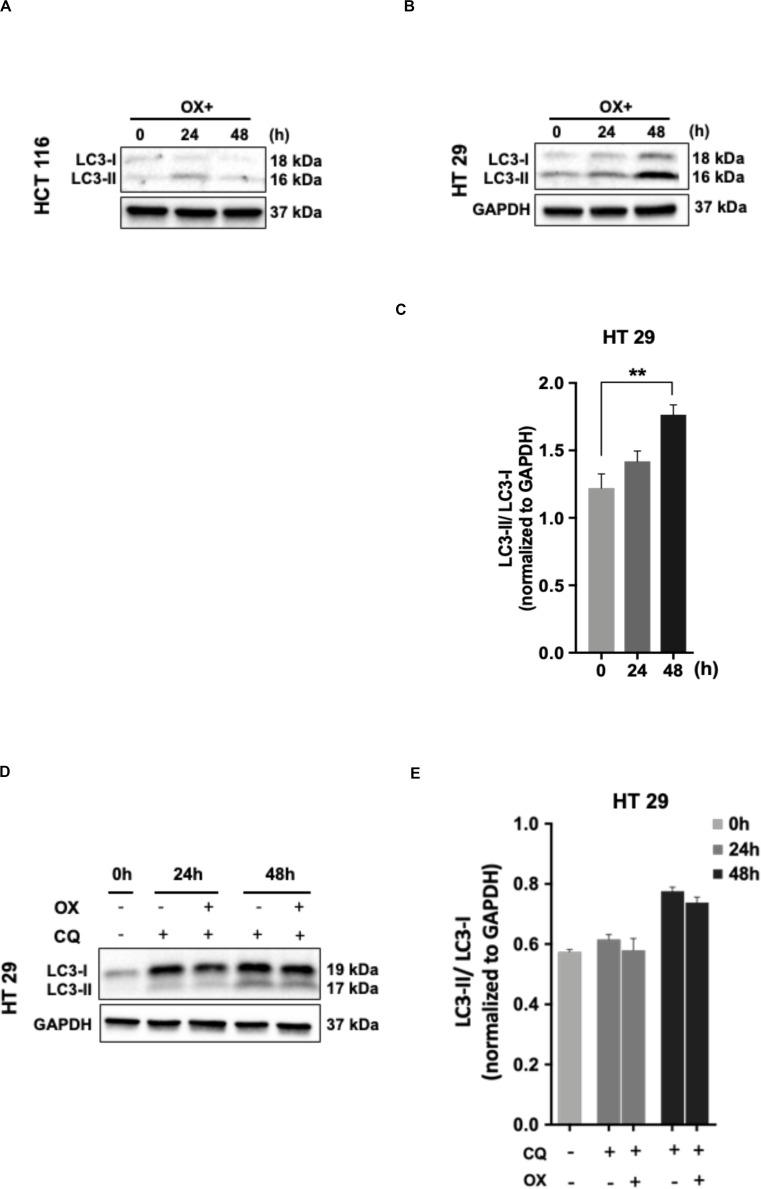
Autophagy as a cytoprotective mechanism of impaired sensitivity to OX in HT 29 cells. **(A,B)** Western blot images showing the conversion of LC3-I to LC3-II. GAPDH served as the loading control. **(C)** Relative ratio of LC3-II/LC3-I quantified by densitometry analysis. Significance was calculated using Welch’s *t*-test; **p = 0.001. **(D,E)** Chloroquine diphosphate (CQ) abrogated OX induced conversion of LC3-I to LC3-II. **(D)** Western blot images showing the conversion of LC3-I to LC3-II. GAPDH served as a loading control. **(E)** Relative ratio of LC3-II/LC3-I quantified by densitometry analysis. The images are representative of three independent experiments and data represented as mean ± SD (*n* = 3).

No LC3 conversion was observed in HCT 116 cells with OX treatment (Figure 5A). However, this conversion was clearly found in HT 29 cells with a time-dependent concomitant increase in LC3-II expression being observed in response to OX treatment (Figure 5B). In HT 29 cells, the accumulation of LC3-II protein was found to be significant after 48 h of OX treatment (Figure 5C). The bar plots represent the LC3-II/LC3-I ratio normalized to the loading control. In the context of p53 status of the colon cancer cells, it was interesting to note that, autophagy was found to be induced only in mutant p53-harboring HT 29 cells in response to OX treatment. The growth inhibition in mutant p53-harboring HT 29 cells as a result of the OX treatment was found to be significantly low as evidenced by initial cytotoxicity assays. Taken together, the impaired sensitivity to OX displayed by the mutant p53-HT 29 cells could be attributed to autophagy being induced in these cells as a cytoprotective mechanism against the genotoxic stress.

In order to confirm the cytoprotective mechanism adopted by mutant p53-HT 29 cells upon OX treatment, the cells were pre-treated with chloroquine diphosphate, a known inhibitor of autophagy which blocks the autophagosome to lysosome fusion (33). Interestingly, chloroquine retracted the OX induced conversion of LC3-I to LC3-II in HT 29 cells in a time-dependent fashion as observed in Figures 5D,E. This further confirms autophagy as a cytoprotective mechanism being attributed to the impaired sensitivity of HT 29 cells to OX treatment.

### Cancer Stemness and β-Catenin Accumulation Attribute Mutant p53 Colon Cancer Cells Impaired Sensitivity to Oxaliplatin

**FIGURE 6:**
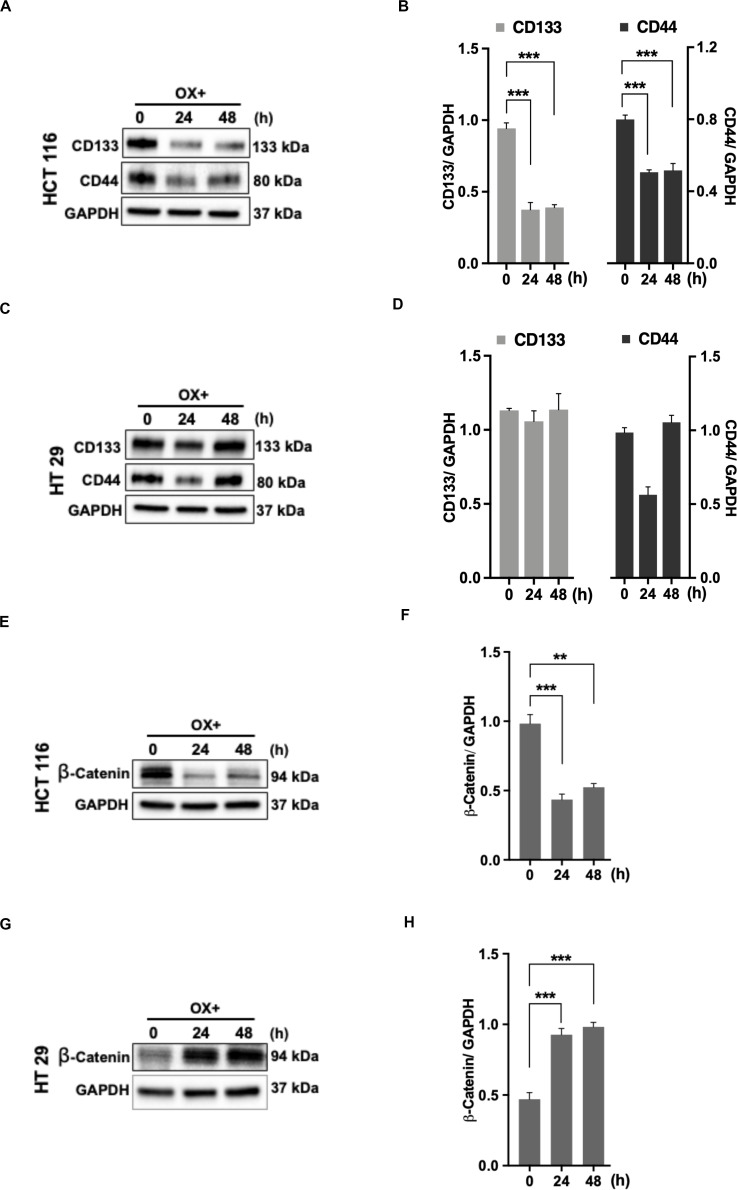
Stemness and β-catenin accumulation contributes to impaired sensitivity of HT 29 cells to OX. **(A,C,E,G)** Western blot images showing the expression of stemness markers CD133, CD44, and β-catenin in HCT 116 cells **(A,E)** and HT 29 cells **(C,G)**, respectively. GAPDH served as the loading control. **(B,D,F,H)** Relative protein levels quantified by densitometry analysis for CD133, CD44, and β-catenin in HCT 116 cells **(B,F)**; in HT 29 cells **(D,H)**, respectively. Significance was calculated using Welch’s *t*-test; **p < 0.0002, ***p < 0.0009. The images are representative of three independent experiments and data represented as mean ± SD (*n* = 3).

The cancer stem cells (CSC) characterized by their self-renewal capability known for their dominated role in chemoresistance have been suggested to be linked to p53 status as well (34). In accordance to this, we looked for the expression of stemness markers in both the colon cancer cells (with differential p53 status) in response to OX treatment. The stemness markers such as CD133 and CD44 were found to be significantly down-regulated with OX treatment in HCT 116 cells (Figures 6A,B) while, no modulated expression of stemness markers with OX treatment was observed in HT 29 cells (Figures 6C,D). This apparently points to the presence of CSC in mutant p53-HT 29 cells which could be attributed to their impaired sensitivity to OX as cancer stemness is a known factor for chemoresistance.

CD44 being a Wnt/β-catenin target gene, along with other studies indicating the link between Wnt/β-catenin signaling pathway, CSC and chemoresistance (35, 36), we determined the expression of β-catenin. In HCT 116 cells, OX treatment was found to inhibit the accumulation of β-catenin (Figures 6E,F) while in HT 29 cells a significant accumulation of β-catenin was observed with OX treatment (Figures 6G,H). The accumulation of β-catenin along with stemness could be attributed to mutant p53 bearing HT 29 cells impaired sensitivity to OX.

## Discussion

Colorectal cancer is one of the highest ranked cancer types in the world with its increasing incidence and mortality rates despite the advancements in cancer therapeutics (1). Over 50% of human CRCs are reported to have defective p53 expression ensued by a mutated *TP53* gene and this defect often contributes to drug resistance, challenging the clinicians in devising chemotherapeutic strategies for management of cancer patients with this mutation (15, 37). In normal cells, p53 expression is induced in response to stressful conditions and the p53 activation indeed induce cell death *via* processes such as cell cycle arrest, DNA repair, senescence or apoptosis (38–40).

In the case of cancers, cells bearing wild-type *TP53* gene expressing functional p53 protein is regarded to be a strong tumor suppressor while, mutated *TP53* gene expressing a mutant p53 protein with GOF tactically overcomes the cell death pathways and make cells resistant to chemotherapeutic drug treatments (41, 42). The p53 mutations being one of the most frequently occurring mutations in colon cancer with its proven role in inducing resistance to therapeutic drugs, the mutant p53 marks itself as a prominent predictive molecular marker for chemoresistance (43–45). Hence, unraveling the mechanisms by which mutant p53 harboring cancer cells evade the cellular death processes induced by a genotoxic stress *via* chemoresistance is of clinical priority, so as to develop strategies that could enhance sensitization of these cells to various chemotherapeutics.

The current study was aimed to investigate the cytotoxic effects induced by the chemotherapeutic drug OX in wild-type *TP53* harboring HCT 116 and mutant *TP53* harboring HT 29 colon cancer cells as well as to elucidate the underlying molecular mechanisms in correlation to their p53 status.

In our study, the HCT 116 cells 48 h posttreatment with OX resulted in 70% growth inhibition, while the growth inhibition in HT 29 cells was significantly reduced accounting to only 40% (Figure 1A). This implied that wild-type p53-HCT 116 cells were sensitive to OX treatment while, the mutant p53-HT 29 cells exhibited an impaired sensitivity to the drug. The wild-type p53 expression is functionally renowned to be a tumor suppressor and is known to induce cell death *via* cell cycle arrest or apoptosis (46). While the mutant p53 accumulation and its GOF is reported to favor tumorigenesis by acquiring oncogenic properties such as survival, proliferation, migration and resistance to drugs (47).

In this study, although OX induced apoptosis in both the colon cancer cell lines after 48 h of treatment, the total apoptosis was comparatively higher in HCT 116 cells than HT 29 cells. As the wild-type p53 expression is known to be a transactivator of the Bax gene (48), we attempted to evaluate the expression of pro-apoptotic “Bax” gene product. The ratio of “Bax/Bcl-2,” a positive indicator of apoptotic process was estimated and we found the ratio to be significantly increased with the OX treatment in HCT 116 cells while, this ratio remained unaltered in HT 29 cells with OX treatment.

Studies have shown that a low Bax/Bcl-2 ratio can favor tumor survival as well as impart resistance to various kinds of cell death stimuli including genotoxic, radiation or hypoxia (49). Moreover, it has been reported that wild-type p53 protein is capable of binding to the promoter of BAX gene and transactivates its expression while, a mutant p53 protein lacks this functionality (24). Taken together, our data indicates that, the wild-type p53 in HCT 116 cells has transactivated the expression of Bax in HCT 116 cells making them sensitive to OX. The mutant p53 in HT 29 cells lacking transactivation function, along with the endogenous expression of anti-apoptotic protein Bcl-2 could be attributed to the impaired sensitivity of these cells to OX. Hence, our study implies the link between p53 status and the Bax/Bcl-2 protein expression ratio in determining the sensitivity of colon cancer cells to the genotoxic stress induced by OX.

The functional mechanisms of wild-type p53 expression is known to be mediated by the activation of its primary effector target p21 – a cyclin-dependent kinase inhibitor (CDKi) (26). Hence, we further investigated the expression profile of p21 protein in both the colon cancer cell types. Interestingly, we found a concomitant increase in the expression of p21 along with p53 after OX treatment in HCT 116 cells (Figures 3A,B) while, in HT 29 cells the OX treatment did not induce any p21 expression wherein, the p53 expression was observed to be constitutive rather than inducive (Figures 3C,D). These findings indicate that, OX induced apoptosis in wild-type p53-HCT 116 cells was mediated by p53/p21 pathway. The possible mechanism being that, the genotoxic stress by OX have induced wild-type p53 expression followed by p21 expression and the p53/p21 complex subsequently mediated the expression of Bax leading to apoptosis. The endogenous expression of mutant p53 protein in HT 29 cells, it’s accumulation in the absence of p21 along with the GOF with oncogenic features could be attributed to the impaired sensitivity to OX displayed by these cells.

Our data also indicated that in HCT 116 cells OX treatment inhibited the expression of anti-apoptotic protein-Bcl-2 where in, both p53 and p21 expression were induced by drug treatment (Figure 2D). In HT 29 cells OX treatment induced the expression of Bcl-2, with no p21 expression, while p53 expression was found to be endogenous (Figure 2F). A study by Kim et al. (50) has shown Bcl-2 as a target for both p53 and p21, as well as the p53/p21 complex together to regulate cancer cell invasion and apoptotic cell death by targeting Bcl-2 protein. Hence, our data further confirms the functional role of wild-type p53 to form p53/p21 complex inhibiting the expression of anti-apoptotic protein Bcl-2 leading to sensitization of HCT 116 cells by OX. While, in HT 29 cells, the endogenous expression of mutant p53, with no functional p53/p21 complex formation along with Bcl-2 expression could have attributed to the impaired sensitivity of these cells to OX.

The p53-dependent-p21 expression mediated growth inhibition could be ascribed to its mechanistic role either as “cell cycle regulator” inducing arrest of cells in G1 or G2 phase (51, 52) or “pro-apoptotic regulator” inducing apoptosis by upregulation of Bax (53). In accordance to this, we performed cell cycle analysis of both the colon cancer cell lines treated with OX using flowcytometry. Apparently, no significant cell cycle arrest in G1 or G2 phase was observed in any of these cell lines with the OX treatment (data not shown). All these data collectively suggest that, the significant growth inhibition of HCT 116 cells (harboring wild-type *TP53*) resulted from OX treatment was due to induction of apoptosis mediated by p53/p21-Bax pathway.

Posttranslational modifications including phosphorylation, acetylation, ubiquitination, methylation, and sumoylation of p53 protein are known to regulate its function inside a cell (54). Among them phosphorylation forms the key modification that stabilizes the p53 protein and thereby activates its functional role as a tumor suppressor in response to cellular stress or DNA damage (55). The stabilization and activation of p53 in response to platinum-based drug treatments (DNA-damaging) are reported to be facilitated through the phosphorylation of N-terminus serine residues especially Ser15, Ser20, and Ser37 by reducing p53 affinity to its negative regulator Hdm2 (29, 56).

In our study, we found a significant increase in the phosphorylation levels of p53 protein at serine residues: Ser15, Ser46, and Ser392 in HCT 116 cells 48 h posttreatment with OX compared to the untreated as identified using phosphoproteome profiler antibody array analysis (Figures 4A,B). While in HT 29 cells, the mutant p53 was found to be constitutively phosphorylated at serine residues: Ser15, Ser46, and Ser392 with no differential phosphorylation levels being observed between untreated *vs.* OX treated cells as evident from the phosphoproteome profiler arrays (Figures 4C,D).

Previous studies have evidenced that in wild-type p53, phosphorylation of N-terminus serine residues: Ser15, 20, and 37 occurs in response to DNA-damaging stresses such as gamma/UV irradiation and metal cadmium (57–59). This phosphorylation in turn is believed to loosen the affinity of p53-Hdm2 interaction leading to stabilization and activation of p53. The phosphorylation of Ser46 of wild-type p53 has been reported to be critical for the induction of apoptosis *via* transactivation of pro-apoptotic protein p53AIp1 (p53-regulated apoptosis-inducing protein 1) (60). An *in vivo* study by Feng et al. (61) has demonstrated the physiological role of Ser46 phosphorylation in regulating apoptosis in mice. The study showed that Ser46-p53 mutant mice had reduced transcription rates of certain pro-apoptotic genes resulting in compromised apoptosis compared to knock-in mice expressing wild-type human *TP53* gene.

The serine residue Ser392 forms one of the most conserved phosphorylation sites in C-terminus of p53 protein and its phosphorylation is believed to facilitate the DNA binding capability of p53 *in vitro* (62). It has been reported that DNA-damaging signals including IR and UV radiation can induce phosphorylation on this site of wild type p53 leading to transactivation of its apoptotic function (63, 64). While, this phosphorylation event has been reported to promote the tumor suppressive function of p53 (65), lack of phosphorylation at Ser389 (serine residue in mice equivalent to human Ser392) of p53 protein has led to the development of bladder tumor in mice (66).

Our study indicates that chemosensitivity of colon cancer cells to OX is dependent on their p53 status and the phosphorylation of N-terminus: Ser15, Ser46 as well as the C-terminus: Ser392. In wild-type p53 this contributes to the transactivation of the apoptotic function of p53 making the cells sensitive to OX. This is evident from the sensitization of wild-type p53 bearing HCT 116 cells to OX being achieved *via* increased phosphorylation of p53 at the aforementioned 3 serine residues, leading to the activation of p53 and mediating apoptosis. The mutant p53 in HT 29 cells with its constitutive phosphorylation at Ser15, Ser46, and Ser392 and accumulation, with no transactivation function but with GOF enriched oncogenic properties could be attributed to the impaired sensitivity of these cells to OX.

We demonstrate for the first time that, endogenous phosphorylation of C-terminus serine residue Ser392 of mutant p53 protein could contribute to the impaired sensitivity of the mutant p53 bearing colon cancer HT 29 cells to OX. This dysregulated phosphorylation would have resulted in a non-activated dysfunctional p53 with reduced DNA-binding activity and hence been less responsive to the DNA-damaging drug-OX. This would fall well in agreement with certain studies showing that Ser392 phosphorylation regulated the oncogenic function of mutant p53 (67) as well as the Ser392 hyper-phosphorylation correlated to poor prognosis with tumor stage and tumor grade in p53-positive cancers (68, 69).

Autophagy an intracellular self-degradative process, forms a mechanism responsible for recycling cellular metabolic substances to maintain homeostasis, while to ascertain its role as antitumor, or tumorigenic is dependent on cellular context (70, 71). It is known that in cancerous cells, autophagy forms an alternative cellular source of energy under metabolic stress and thereby adapts to a smarter response with cancer therapies (30, 72). Certain studies suggests that autophagy can act as a tumor-suppressive factor in case of solid tumors, for instance in thyroid cancer (73), and also can increase cancer cell chemo-sensitivity even in hematological malignancy such as lymphoma (74). However, in disparity, a number of study findings support the role of autophagy in facilitating tumorigenesis by attenuating drug induced cytotoxicity augmenting chemoresistance in solid tumors (75–77) as well as in hematological malignancies (70, 78). In general, autophagy is doomed to be an adaptive response to stressful conditions such as starvation or hypoxia in tumors wherein it forms a pro-survival mechanism promoting tumor cell survival (79). Whilst under genotoxic stress induced by chemotherapeutic drugs, autophagy forms a cytoprotective mechanism in cancer cells favoring chemoresistance (80).

In our study, the conversion of LC3-I protein to LC3-II: a hallmark of autophagy induction was observed with OX treatment in HT 29 (mutant p53) colon cancer cells (Figures 5B,C). A time-dependent accumulation of LC3-II protein with OX treatment was observed in HT 29 cells while, no such accumulation of LC3-II protein was found in HCT 116 cells indicating no induction of autophagy in response to OX treatment (Figure 5A).

Many studies have described the role of autophagy in tumor chemoresistance as context-dependent (42) suggesting that wild-type p53 induces autophagy (81), whereas mutant p53 represses (82, 83) it. However, studies depicting autophagy as a cytoprotective mechanism of chemoresistance in colon cancer cells being linked their p53 status are limited or nearly nil. In this context, our findings demonstrate for the first time, “autophagy” as a possible cytoprotective mechanism for impaired sensitivity of mutant p53 harboring HT 29 colon cancer cells to OX.

There is accumulating evidence that exists on the drug-resistant property exhibited by cancerous cells harboring mutant p53 (15, 84, 85). Studies also indicate that the oncogenic properties such as tumorigenesis, anti-apoptotic as well as drug resistance featured by certain cancer cell types can be attributed to the presence of CSC as well as mutant p53 protein accumulation (34, 86). CSCs are known to be potentially resistant to chemotherapy and hence, pose the risk of disease recurrence as the conventional chemotherapies mostly fails to eliminate the CSC (33, 87). Many previous reports have also suggested the possible link between CSC and p53 status, with CSC markers such as CD133 and CD44 being repressed in wild-type p53 while, their expression contributing to the CSC characteristics in various mutant p53 cancer cell types (88, 89).

In the current study, we found that cancer specific stemness markers: CD133 and CD44 in wild-type p53-HCT 116 cells were significantly down-regulated in their expression with OX treatment (Figures 6A,B). While in mutant p53 HT 29 cells, OX treatment did not modulate the expression of stemness marker proteins (Figures 6C,D). This perhaps indicates the existence of a subpopulation of cancer cells with self-renewal capability and these CSCs along with the accumulation of mutant p53 protein (as shown before) could be attributed to the impaired sensitivity to OX displayed by HT 29 cells.

In line with the emerging evidences highlighting the association of cancer stemness and chemotherapy resistance (90–93), various studies have also underpinned the link between Wnt/β-catenin signaling pathway, CSC and chemoresistance. For instance, Urushibara et al. (35) tried to inhibit the expression of CSC proteins using a Wnt/β-catenin signaling inhibitor “IC-2” and found enhanced cytotoxicity to the drug 5-FU (fluorouracil) by colon cancer cells. Another study by Chen et al. (36) has shown that suppression of Axin2 (a negative feedback regulator of Wnt/β-catenin signaling) by micro RNA: miR-103/107 enhanced chemoresistance of colon cancer cells *via* inducing stemness.

A significant accumulation of β-catenin in response to OX treatment in mutant p53-HT 29 cells revealed the involvement of Wnt/β-catenin pathway in attributing HT 29 cells impaired sensitivity to OX. In contrast, this accumulation was not observed in wild-type p53-HCT 116 cells which were sensitive to OX.

Though studies have reported the role of high Wnt activity as well as the involvement of Wnt/β-catenin signaling in regulating CSC properties (94–96), to our knowledge our study forms the first ever report demonstrating cancer stemness and Wnt/β-catenin signaling as factors contributing to the impaired sensitivity of the mutant p53 harboring colon cancer HT 29 cells to OX.

**FIGURE 7:**
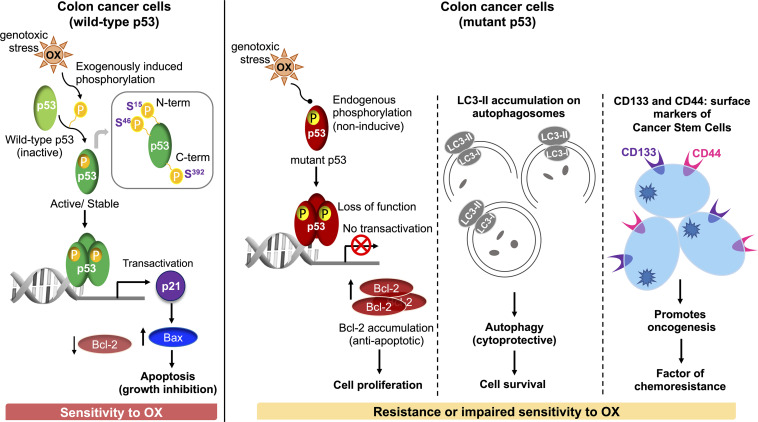
Schematic representation of molecular mechanisms attributing to the sensitivity of wild-type p53-harboring colon cancer cells to OX *vs.* resistance or impaired sensitivity of the mutant p53-harboring colon cancer cells to OX.

In conclusion, our study demonstrates for the first time that dysregulated phosphorylation of the N-terminus and C-terminus serine residues of mutant p53 protein critically contributes to impaired sensitivity of mutant p53-harboring HT 29 colon cancer cells to OX (Figure 7). Our findings also demonstrate for the first time, autophagy as a cytoprotective mechanism for impaired sensitivity towards OX in mutant p53-harboring colon cancer cells. Our study also suggests cancer stemness and Wnt/β-catenin signaling as factors contributing to the impaired sensitivity of mutant p53-harboring colon cancer cells to OX which may have therapeutic value for treating colon cancers with OX in future.

## Data Availability Statement

The raw data supporting the conclusions of this article will be made available by the authors, without undue reservation.

## Author Contributions

RK conceptualized and designed the study. SU and MS mentored the study. LT, JH, and FS performed the experiments. RK, LT, and KS analyzed the data. LT, JH, and RK made the figures. RK created the illustration. LT, JH, and RK wrote the manuscript with comments and input from all co-authors. MK and JB provided linguistic support. All authors have read and approved the final version of manuscript.

## Conflict of Interest

The authors declare that the research was conducted in the absence of any commercial or financial relationships that could be construed as a potential conflict of interest.
