# Supplementary material for: Dysregulated Phosphorylation of p53, Autophagy and Stemness Attributes the Mutant p53 Harboring Colon Cancer Cells Impaired Sensitivity to Oxaliplatin
Source: Front Oncol. 2020 Aug 28;10:1744. doi: 10.3389/fonc.2020.01744 (PMC7485421; doi:10.3389/fonc.2020.01744)
Supplement: Supplementary file 1 [file Data_Sheet_1.PDF]

Supplementary Material

Supplementary Figure

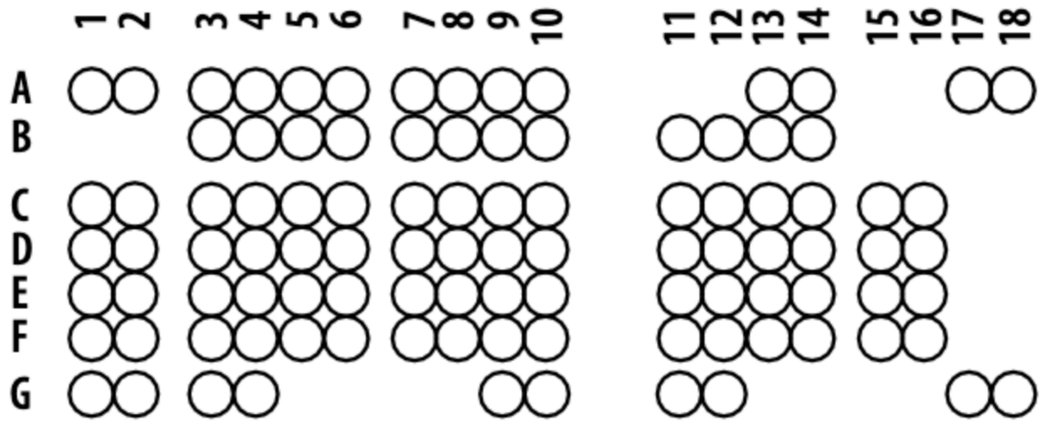

Supplementary Figure 1. Human Proteome Profiler Phospho-Kinase Array (R&D) coordinates. Each antibody is spotted in duplicates in the membrane and the corresponding target protein names and the phosphorylation sites are as below.

Membrane/Coordinate	Target/Control	Phosphorylation Site
A-A1, A2	Reference Spot	---
A-A3, A4	p38α	T180/Y182
A-A5, A6	ERK1/2	T202/Y204, T185/ Y187
A-A7, A8	JNK1/2/3	T183/Y185, T221/ Y223
A-A9, A10	GSK-3α/β	S21/S9
B-A13, A14	p53	S392
B-A17, A18	Reference Spot	---
A-B3, B4	EGF R	Y1086
A-B5, B6	MSK1/2	S376/S360
A-B7, B8	AMPKα1	T183
A-B9, B10	Akt 1/2/3	S473
B-B11, B12	Akt 1/2/3	T308
B-B13, B14	p53	S46

A-C1, C2	TOR	S2448
A-C3, C4	CREB	S133
A-C5, C6	HSP27	S78/S82
A-C7, C8	AMPKα2	T172
A-C9, C10	β-Catenin	---
B-C11, C12	p70 S6 Kinase	T389
B-C13, C14	p53	S15
B-C15, C16	c-Jun	S63
A-D1, D2	Src	Y419
A-D3, D4	Lyn	Y397
A-D5, D6	Lck	Y394
A-D7, D8	STAT2	Y689
A-D9, D10	STAT5a	Y694
B-D11, D12	p70 S6 Kinase	T421/S424
B-D13, D14	RSK1/2/3	S380/S386/S377
B-D15, D16	eNOS	S1177
A-E1, E2	Fyn	Y420
A-E3, E4	Yes	Y426
A-E5, E6	Fgr	Y412
A-E7, E8	STAT6	Y641
A-E9, E10	STAT5b	Y699
B-E11, E12	STAT3	Y705
B-E13, E14	p27	T198
B-E15, E16	PLC-γ1	Y783
A-F1, F2	Hck	Y411
A-F3, F4	Chk-2	T68
A-F5, F6	FAK	Y397
A-F7, F8	PDGF Rβ	Y751
A-F9, F10	STAT5a/b	Y694/Y699
B-F11, F12	STAT3	S727
B-F13, F14	WNK1	T60
B-F15, F16	PYK2	Y402
A-G1, G2	Reference Spot	---
A-G3, G4	PRAS40	T246
A-G9, G10	PBS (Negative Control)	---
B-G11, G12	HSP60	---
B-G17, G18	PBS (Negative Control)	---
